# The Prevalence of Advanced Interatrial Block and Its Relationship to Left Atrial Function in Patients with Transthyretin Cardiac Amyloidosis

**DOI:** 10.3390/jcm10132764

**Published:** 2021-06-23

**Authors:** Thomas Lindow, Per Lindqvist

**Affiliations:** 1 Kolling Institute, Royal North Shore Hospital, University of Sydney, Sydney, NSW 2065, Australia; 2 Department of Clinical Physiology, Research and Development, Växjö Central Hospital, Region Kronoberg, 351 88 Växjö, Sweden; 3 Clinical Physiology, Clinical Sciences, Lund University, 221 00 Lund, Sweden; 4 Department of Clinical Physiology, Surgical and Perioperative Sciences, Umeå University, 901 87 Umeå, Sweden; per.lindqvist@umu.se

**Keywords:** interatrial block, left atrial strain, cardiac amyloidosis, transthyretin amyloid

## Abstract

Background: Advanced interatrial block (aIAB), which is associated with incident atrial fibrillation and stroke, occurs in the setting of blocked interatrial conduction. Atrial amyloid deposition could be a possible substrate for reduced interatrial conduction, but the prevalence of aIAB in patients with transthyretin cardiac amyloidosis (ATTR-CA) is unknown. We aimed to describe the prevalence of aIAB and its relationship to left atrial function in patients with ATTR-CA in comparison to patients with HF and left ventricular hypertrophy but no CA. Methods: The presence of aIAB was investigated among 75 patients (49 patients with ATTR-CA and 26 with HF but no CA). A comprehensive echocardiographic investigation was performed in all patients, including left atrial strain and strain rate measurements. Results: Among patients with ATTR-CA, 27% had aIAB and in patients with HF but no CA, this figure was 21%, (*p* = 0.78). The presence of aIAB was associated with a low strain rate during atrial contraction (<0.91 s^−1^) (OR: 5.2 (1.4–19.9)), even after adjusting for age and LAVi (OR: 4.5 (1.0–19.19)). Conclusions: Advanced interatrial block is common among patients with ATTR-CA, as well as in patients with heart failure and left ventricular hypertrophy but no CA. aIAB is associated with reduced left atrial contractile function.

## 1. Introduction

**Figure 1:**
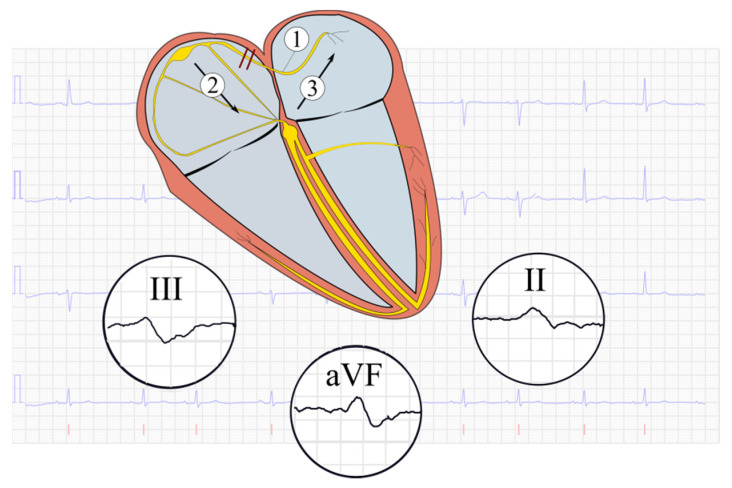
Description of the atrial depolarization route in advanced interatrial block with zoomed images of the P waves in inferior leads (II, aVF and III), in the case of a block in Bachmann’s bundle (1). Normally, depolarization of the atria travels through the right and left atria through internodal tracts, e.g., Bachmann’s bundle. Since impulse propagation from the right atrium to the left atrium is blocked, the right atrium will be depolarized first (2) followed by a retrograde depolarization of the left atrium (3), resulting in biphasic P waves in inferior leads. Modified from [10], reprinted with permission.

During atrial depolarization, the electrical impulses are propagated from the right to the left atrium through several conduction pathways, including inter-nodal tracts between the atria, e.g., Bachmann’s bundle [1]. In the presence of a block in Bachmann’s bundle, interatrial block (IAB) may occur. This block can occur with varying grades, from partial IAB resulting in increased P wave duration only, to advanced IAB (aIAB) in which P wave duration is increased (≥120 ms) and a biphasic P wave pattern occurs in inferior leads (II, III, aVF) [2,3,4,5]. In case of a complete block in Bachmann’s bundle, depolarization will follow a caudo-cranial route, in which the right atrium is depolarized first (caudal route) followed by a retrograde depolarization of the left atrium (cranial route, Figure 1) [6], which explains the biphasic P wave pattern in inferior leads. 

IAB in combination with supraventricular arrhythmias is known as Bayes’ syndrome [7]. aIAB, but not partial IAB, has been shown to be a significant predictor of new-onset and recurrent AF [8]. Furthermore, IAB is highly prevalent in patients with embolic stroke [7,9,10]. Due to the retrograde and delayed depolarization of the left atrium, poor left atrial electro-mechanical function is plausible, and this may predispose clot formation [9]. 

Transthyretin cardiac amyloidosis (ATTR-CA) has received increased attention in recent years, and prevalence of the disease has been reported to be markedly higher than previously expected [11]. For example, approximately one in eight patients undergoing transcatheter aortic valvular replacement [12] and about one in five patients with left ventricular hypertrophy and heart failure (HF) could be diagnosed with ATTR-CA [13]. ATTR-CA is nowadays an important clinical reality for both cardiologists [11] and radiologists [14]. In ATTR-CA, deposition of transthyretin (TTR) amyloid fibrils occurs in the extracellular space of the heart, affecting not only the ventricles but the atria, as well as resulting in impaired ventricular and atrial function [15,16,17]. In addition to HF, ATTR-CA is associated with increased risk of arrhythmias, in the form of both conduction abnormalities, such as bundle branch block or AV block, and atrial fibrillation [18,19,20]. It is plausible that conduction within the atrium, as well as between the atria, is affected by ATTR-CA, and that the prevalence of aIAB may therefore be increased in this population. Since both stroke and atrial fibrillation are common among patients with ATTR-CA [21,22], it is important to identify reliable predictors of such adverse events.

Our primary aim was to describe the prevalence of aIAB in patients with ATTR-CA in comparison to patients with HF and left ventricular hypertrophy but no CA. A secondary aim was to evaluate the association between aIAB and left atrial function.

## 2. Material and Methods

We performed an observational study including patients with increased left ventricular wall thickness and HF, previously included in a separate study on ATTR-CA [13]. In that study, out of 2238 patients with HF, cardiomyopathy or hypertensive heart disease based on hospital records (International Classification of Diseases 10 (ICD-10): I50, I42, I43 or I11), 174 patients had interventricular septal thickness (IVSd) exceeding 14 mm on echocardiography after re-evaluation of all patients reported to have IVSd ≥ 11 mm. All patients were diagnosed for ATTR-CA by ^99m^Tc-3,3-diphosphono-1,2-propanodicarboxylic acid (^99m^Tc-DPD) scintigraphy, a reliable method for diagnosing ATTR-CA without the need for histology [23]. DPD scans were graded according to the Perugini classification (grade 0: no cardiac uptake with normal bone uptake (i.e., negative); grades 1 to 3: increasing cardiac uptake with increasing bone attenuation) [24]. Excluding amyloid light-chain (AL) amyloidosis in all patients was based on blood and urine analysis of serum-free light-chain abnormalities (Freelite, Binding Site reagent, reference range 0.27–1.64) and the absence of monoclonal bands. Patients with abnormalities in these analyses were carefully evaluated and their clinical history and disease progression reviewed to assess the possibility of them having AL amyloidosis. Sequencing of the TTR-gene was also undertaken in all patients to diagnose ATTRv (v for variant) amyloidosis. From this cohort, we found 11 patients with ATTRwt (wt for wild-type) and 26 patients with HF but no CA, all in sinus rhythm.

In addition, we retrospectively included 38 patients with ATTRv from our local database, all diagnosed with ATTR-CA based on positive DPD scintigraphy (grades 2–3) and genetic testing. All patients were in sinus rhythm.

### 2.1. Electrocardiography

Twelve-lead ECGs were recorded using computerized electrocardiographs (GE system Mac 5000/5500, General Electric, Chicago, IL, USA). Blinded ECG analysis was performed for all patients (TL). The ECG criteria for aIAB were P wave duration ≥ 120 ms and biphasic P waves in leads II, III and aVF [6]. Atypical aIAB was defined as P wave duration ≥120 ms and biphasic P waves in leads III and aVF and an isodiphasic final component in lead II; or P wave duration ≥120 ms and biphasic P waves in leads III and aVF and a triphasic P wave in lead II; or P wave duration ≥120 ms and a biphasic P wave in lead II and isodiphasic P waves in leads III and aVF; or P wave duration ≤120 ms but typical biphasic P waves in leads II, III and aVF [25].

### 2.2. Echocardiography

A comprehensive transthoracic echocardiographic exam was performed in all patients using a Vivid E9 system (GE Medical Systems, Horten, Norway) equipped with an adult 1.5–4.3 MHz phased array transducer. Offline analyses were performed using commercially available software (General Electric, EchoPac version BT 13, 113.0, Waukesha, WI, USA). Echocardiograms were digitally stored and were reanalyzed by an experienced operator (P.L.) blinded to the clinical diagnosis. Doppler variables to quantify left ventricular diastolic function were measured according to standard echocardiographic methods [26]. Left atrial (LA) measurements, including LA volume (LAV, LAV indexed to body surface area ([BSA] LAVi), LA strain and strain rate were measured at ventricular systole (LASs and LASRs) and atrial systole (LASa and LASRa)). When measuring LA strain and strain rate, anatomical landmarks were used and care was taken over echocardiographic image acquisition to ensure adequate LA tracking, avoiding foreshortening of the LA cavity or interference with the pulmonary veins or LA appendage. Longitudinal myocardial deformation, assessed by 2-dimensional echocardiography using speckle tracking, was analyzed offline. From the apical 4-chamber view, a point-and-click technique was used to create a horseshoe-shaped ROI within the LA. The endocardial border of the septal and the roof/apical and lateral walls of the LA were traced manually, in order to analyze global LA strain and strain-rate measurements, respectively. Tracings which did not accurately track LA structures were discarded. Reduced LA booster pump function was defined as strain rate during atrial contraction (SRa) < 0.91^−s^ [27]. 

### 2.3. Statistical Analysis

Continuous variables are described using mean and standard deviation (SD) if normally distributed, otherwise as median and interquartile range (IQR). The Shapiro–Wilk test was used to test for normality. A χ^2^ test was used to assess proportional differences between groups. A Student’s *t*-test or Mann–Whitney U test was used for comparisons of means or medians between groups for normally or non-normally distributed variables, respectively. 

When evaluating the association between aIAB and LA function, no stratification based on ATTR-CA diagnosis was made, i.e., patients were stratified by aIAB despite their underlying condition. Odds ratios for reduced SRa were calculated using a univariate binary logistic regression model. Variables with *p*-values < 0.05 at univariate analysis were entered into a multivariable model. Model improvement was evaluated by area under the curve (AUC). Statistical analysis was performed using IBM SPSS Statistics for Windows, Version 26.0, IBM Corp., Armonk, NY, USA).

## 3. Results

**Figure 2:**
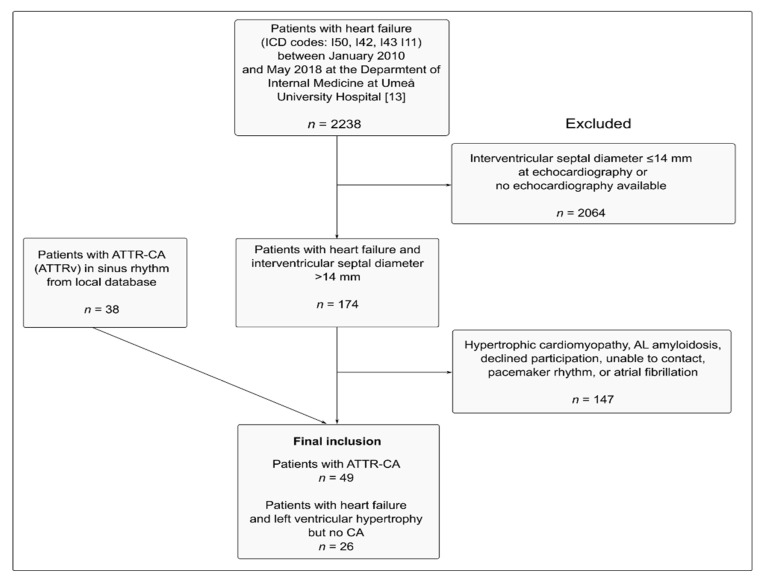
Flowchart of patient inclusion and exclusion. Abbreviations: ICD—International Classification of Diseases; ATTR-CA—transthyretin cardiac amyloidosis, ATTRv—amyloid transthyretin variant; AL—amyloid light-chain.

In total, 75 patients were included (49 patients with ATTR-CA and 26 with HF but no CA) (Figure 2). Baseline characteristics, including echocardiographic characteristics, are presented in Table 1. 

### 3.1. Prevalence of Advanced Interatrial Block in ATTR-CA

Increased P wave duration (≥120 ms) was present in 54 patients (43 (54%) ATTR-CA, 11 (39%) HF no CA, *p* = 0.25). Among patients with ATTR-CA, 8 patients (16%) had typical aIAB and 5 (10%) atypical aIAB. The prevalence of aIAB was not significantly higher in ATTR-CA compared to the reference group (27% vs. 21%, *p* = 0.67). The heart rate was higher among patients with ATTR-CA compared to those without. Left anterior hemiblock was more common among patients with ATTR-CA compared to those without. There was no difference in prevalence of the AV block or left bundle branch block. ECG findings are presented in Table 2. 

### 3.2. Advanced Interatrial Block and Left Atrial Function

**Figure 3:**
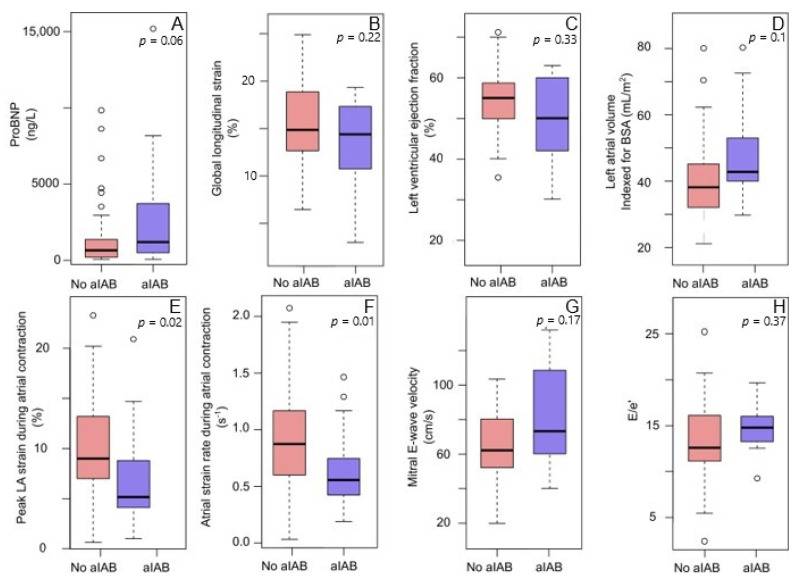
Biomarker and echocardiographic measures in patients with and without advanced interatrial block (aIAB). Patients without aIAB are presented in red bars and patients with aIAB in blue bars. Upper panel: (**A**) N-terminal pro b-type natriuretic peptide (proBNP); (**B**) global longitudinal strain; (**C**) left ventricular ejection fraction; (**D**) left atrial volume indexed for body surface area (BSA); (**E**) peak left atrial (LA) strain during atrial contraction; (**F**) atrial strain rate during atrial contraction; (**G**) mitral E-wave velocity; (**H**) E/e’. Peak left atrial longitudinal strain (**E**) and atrial strain rate (**F**) were significantly reduced in patients with advanced interatrial block. Peak left atrial longitudinal strain (**E**) and atrial strain rate (**F**) were significantly reduced in patients with advanced interatrial block.

aIAB was present in 18 patients (13 with ATTR-CA). There were no statistically significant differences in mean age (79 vs. 76 years, *p* = 0.09), blood pressure (131/79 vs. 134/80 mm Hg, *p* = 0.50 and 0.88), body mass index (25.5 vs. 24.5 kg/m^2^, *p* = 0.44) or the presence of heart failure medication (85% vs. 71 %, *p* = 0.27) between patients with aIAB and patients without aIAB. Biomarker and echocardiographic findings in relation to aIAB are presented in Figure 2. Using strain analysis, there was a greater reduction in atrial function in patients with aIAB compared to those without (Figure 3), both for peak longitudinal LA strain and strain rate. aIAB was associated with low SRa (<0.91 s^−1^) (OR: 5.2 (1.4–19.9)) even after adjusting for age and LAVi (OR: 4.5 (1.0–19.19)) (Table 3). SRa was reduced in 83% of patients with aIAB, compared to 49% among those without (*p* = 0.04). The level of N-terminal pro-brain natriuretic peptides (NT-proBNP) was higher in patients with CA-ATTR and aIAB compared to those without aIAB (median (IQR): 1443 (909, 4344) vs. 942 (262, 2849)), but did not reach statistical significance (*p* = 0.1). 

## 4. Discussion

This is the first study to describe the prevalence of aIAB in patients with ATTR-CA, which we found to be high (27%). Although the possible presence of atrial amyloid deposition was a theoretically appealing substrate for reduced interatrial conduction, the prevalence was not significantly higher than among patients with HF and left ventricular hypertrophy but no CA. Nonetheless, the high prevalence is clinically relevant. Firstly, it may add to the stroke-risk assessment in patients with ATTR-CA. ATTR-CA is associated with a high risk of stroke in itself and independently of conventional risk assessment, such as left atrial size or CHA_2_DS_2_-VASc score [21], and aIAB has been shown to be associated with increased risk of stroke [7,28,29]. Atrial fibrillation is common among patients with ATTR-CA [22], but although atrial fibrillation is a well-established risk factor for stroke, the reported lack of temporal relation suggests that it may not be the direct cause of embolism [30]. Instead, it can be considered (another) risk factor and marker of atrial disease [31]. Identifying additional prognostically relevant markers of atrial disease, both electrocardiographic and echocardiographic ones (e.g., aIAB, LA strain, etc.), is important. Secondly, early identification of atrial fibrillation is important in ATTR-CA. Maintained sinus rhythm has been shown to be associated with improved survival, and strategies (both medical and invasive) are more effective in the earlier stages of the disease [22]. Advanced IAB by resting 12-lead ECG is an easily accessible marker for the identification of patients with a high risk of atrial fibrillation and can be used to indicate which patients should undergo further investigations to detect paroxysmal atrial fibrillation, such as long-term ECG recordings.

In congestive heart failure, the atria are commonly affected by fibrosis [17]. This may explain the high prevalence of aIAB in HF patients. Out of 390 patients with HF undergoing cardiac resynchronization therapy, 38% were found to have aIAB. In that population, aIAB independently predicted the development of new-onset atrial fibrillation [32]. 

In recent years, aIAB has been reported for other populations as well, such as patients treated with transcatheter aortic valve implantation (12%) [33], patients with ST-elevation myocardial infarction (6%) [34] or takotsubo cardiomyopathy (5%) [35], patients with human immunodeficiency virus (4%) [36], etc. 

Lacalzada-Almeida et al. performed an observational prospective study in which they followed 98 patients scheduled for a preoperative ECG before non-cardiac surgery for almost 2 years. Advanced IAB was present in 22% of patients. New atrial fibrillation or stroke occurred in 15% without IAB, and in 41% of those with aIAB. In that study, reduced SRa was the strongest predictor of incident AF or stroke [37]. Nochioka et al. described impaired LA function in patients with different subtypes of CA. LA active emptying function was worse in wild-type ATTR compared to other types of CA [38]. In the present study, we found a significant association between aIAB and reduced LA function, even after adjusting for left atrial size and age.

Amyloid deposition in the atria also occurs in age-related degenerative diseases other than ATTR, and can even occur as an isolated atrial disease [17]. Atrial amyloidosis is recognized as a class IV atrial cardiomyopathy, according to a recent consensus document [17]. Atrial fibrosis resulting in conduction inhomogeneity has been suggested to be an arrhythmogenic substrate [39]. Two decades ago, Röcken et al. obtained tissue samples from the right atrial appendage in patients undergoing cardiac surgery and found that amyloid deposition was independently associated with an increased risk of atrial fibrillation [15]. In another study, it was shown that among patients with ATTR-CA (ATTRv), reduced atrial function can occur even in in the absence of LA dilatation, supposedly due to a limitation of LA distension by amyloid deposition. In that cohort, reduced SRa independently predicted atrial arrhythmia [40]. 

This study is limited by its retrospective design. In addition, although the ECG reader (T.L.) was blinded to the clinical outcome, among patients with imaging evidence of left ventricular hypertrophy, CA can be suspected based on the absence of electrocardiographic findings of left ventricular hypertrophy or reduced QRS amplitudes, and thus blinding could not be considered complete. Nonetheless, T.L. was blinded to the echocardiographic results for all patients. Additionally, echocardiographic measurements were performed by a single (highly experienced) reader only (P.L.). LA strain and strain rate values have proven to be highly reproducible, and SRa in particular had the highest intra- and interobserver agreement [41].

Additionally, the sample size is small and the lack of significant differences in prevalence of aIAB between ATTR-CA and patients with HF but no CA may be due to a type II error. However, the prevalence of aIAB was high among patients with ATTR-CA, a finding that is clinically relevant despite the lack of difference between the two groups. 

## 5. Conclusions

Advanced interatrial block is common among patients with ATTR-CA, but not more common than among patients with HF and left ventricular hypertrophy but no CA. aIAB is associated with reduced LA phasic function independently of age and LA size.

## Figures and Tables

**Table 1:** Baseline and echocardiographic characteristics.

	All Patients	HF No ATTR-CA	ATTR-CA	*p*-Value
n	75	26	49	
Age, years (mean (SD))	75.4 (8.0)	75.8 (7.5)	75.1 (8.5)	0.73
Body mass index (mean (SD))	26.0 (4.8)	29.2 (5.5)	24.9 (4.1)	
Perugini score (%)				<0.001
0	22 (31.9)	22 (100.0)	0 (0.0)	
1	3 (4.3)	0 (0.0)	3 (6.4)	
2	18 (26.1)	0 (0.0)	18 (38.3)	
3	26 (37.7)	0 (0.0)	26 (55.3)	
NT-ProBNP, ng/L (median (IQR))	782 (231, 1616)	408 (1216, 5440)	844 (241, 1640)	0.42
Troponin T, ng/L (median (IQR))	25 (16, 40)	22 (13, 34)	30 (19, 45)	0.27
Systolic BP, mm Hg (mean (SD))	137.6 (20.0)	145.4(23.2)	133.3 (16.9)	0.01
Diastolic BP, mm Hg (mean (SD))	81.3 (10.2)	85.6 (11.4)	78.9 (87)	0.006
Heart failure medications (*n*, %)	52 (69.3)	25 (96.2)	27 (55.1)	0.001
LVDD, mm (mean (SD))	44.6 (6.4)	49.2 (5.8)	42.1 (5.5)	<0.001
IVSD, mm (median (IQR))	16 (15, 20)	16(15, 17)	18 (15, 21)	0.067
PWT, mm (median (IQR))	13 (10, 14)	11 (9, 12)	13 (12, 15)	<0.001
LVEF, % (median (IQR))	55 (46, 58)	55 (46, 60)	50 (46, 55)	0.20
E, cm/s (mean (SD))	68.26 (21.41)	58.68 (20.25)	73.36 (20.43)	0.005
e’, cm/s (median (IQR))	4.0 (3.8, 6.0)	4.0 (3.0, 5.0)	5.0 (4.0, 6.0)	0.07
E/e’ (median (IQR))	14.9 (12.8, 16.7)	14.0 (11.3, 16.2)	15.2 (13.3 18.3)	0.31
GLS, % (mean (SD))	14.1 (4.7)	13.2 (4.6)	14.6 (4.7)	0.21
Relative wall thickness (median (IQR))	0.46 (0.38, 0.55)	0.41 (0.38, 0.48)	0.60 (0.52, 0.75)	0.01
LAVi, mL/m^2^ (mean (SD))	40.54 (16.61)	41.00 (18.80)	39.38 (10.13)	0.82
Tricuspid regurgitant velocity, cm/s (mean (SD))	26.9 (8.3)	28.1 (9.5)	26.4 (7.9)	0.54
Peak atrial longitudinal strain, % (median (IQR))	12.20 (7.57, 16.05)	10.30 (6.45, 14.95)	13.80 (8.55, 18.50)	0.17
Peak atrial longitudinal strain (atrial contraction), % (median (IQR))	8.32 (5.15, 12.15)	8.16 (6.10, 10.75)	8.32 (5.00, 14.00)	0.74
Atrial systolic strain rate, s^−1^ (median (IQR))	0.80 (0.57, 1.20)	0.74 (0.65, 0.96)	0.80 (0.55, 1.94)	0.38
Atrial strain rate (atrial contraction), s^−1^ (median (IQR))	0.81 (0.52, 1.20)	0.88 (0.57, 1.20)	0.81 (0.46, 1.10)	0.64

Abbreviations: HF—heart failure; ATTR-CA—transthyretin cardiac amyloidosis; SD—standard deviation; ProBNP—N-terminal pro b-type natriuretic peptide; BP—blood pressure; LVDD—left ventricular diastolic diameter; IVSD—interventricular systolic diameter; PWT—posterior wall thickness; LVEF—left ventricular ejection fraction; GLS—global longitudinal strain; IQR—interquartile range; LAVi—indexed left atrial volume.

**Table 2:** ECG findings stratified by ATTR diagnosis.

	HF No ATTR-CA	ATTR-CA	*p*-Value
*n*	26	49	
Heart rate, min^−1^ (mean (SD))	64.7 (12.5)	74.8 (11.6)	0.001
P wave duration, ms (mean (SD))	103.1 (20.2)	112.0 (22.0)	0.09
P wave duration ≥120 ms (*n*, %)	10 (38.5)	25 (51.0)	0.43
Advanced IAB (*n*, %)	5 (21.4)	13 (26.5)	0.67
Typical aIAB	4 (15.4)	8 (16.3)	
Atypical aIAB	1 (3.8)	5 (10.2)	
PR interval, ms (mean (SD))	199.8 (37.7)	211.8 (38.4)	0.20
AV-block I, ms (*n*, %)	9 (34.6)	21 (42.9)	0.66
QRS duration, ms (median [IQR])	106 (101, 121)	106 (92, 128)	0.24
Left bundle branch block	3 (11.5)	6 (12.2)	1.0
Left anterior hemiblock	0 (0)	12 (24.5)	0.006
Right bundle branch block	0 (0)	2 (4.1)	0.54
Right bundle branch block and left anterior hemiblock	0 (0)	3 (12.7)	0.04
Pathological Q waves (*n*, %)	2 (7.7)	7 (14.3)	0.48
ST-segment changes (*n*, %)	10 (38.5)	14 (28.6)	0.44

Abbreviations: HF—heart failure; ATTR-CA—transthyretin cardiac amyloidosis; SD—standard deviation; IAB—interatrial block; AV—atrioventricular; IQR—interquartile range.

**Table 3:** Univariate and multivariable analysis.

	Reduced Atrial Strain Rate during Atrial Contraction					
	Univariate Analysis			Multivariable Analysis		
	Odds Ratio (95% CI)	AUC (95% CI)	*p*		AUC (95% CI)	*p*
ATTR-CA	0.98 (0.37–2.56)	0.50 (0.37–0.64)	0.96	-		
P wave duration	1.02 (0.98–1.04)	0.62 (0.49–0.74)	0.10	-		
Advanced interatrial block	5.17 (1.35–19.9)	0.63 (0.52–0.75)	0.02	4.40 (1.00–19.19)	0.76 (0.66–0.89)	0.049
Age	1.08 (1.01–1.16)	0.65 (0.53–0.78)	0.02	1.08 (1.01–1.16)		0.03
LAVI	1.06 (1.01–1.11)	0.70 (0.58–0.82)	<0.01	1.07 (1.01–1.12)		0.01
Sex	0.98 (0.39–2.47)	0.50 (0.37–0.64)	0.97	-		
LVEF	1.0 (0.95–1.06)	0.50 (0.37–0.63)	0.99	-		
Global longitudinal strain	0.92 (0.83–1.02)	0.63 (0.49–0.76)	0.10	-		

Abbreviations: AUC—area under the curve; ATTR-CA—transthyretin cardiac amyloidosis; CI—confidence interval; LAVI—indexed left atrial volume; LVEF—left ventricular ejection fraction.

## Data Availability

The data presented in this study are available on request from the corresponding author. The data are not publicly available due to ethical and privacy reasons.
